# Low Concentrations of Sulfoxaflor Do Not Adversely Affect mRNA Levels in Various Testicular Cells When Administered to Either Mature or Immature Mice

**DOI:** 10.3390/jox15060189

**Published:** 2025-11-07

**Authors:** Hayato Terayama, Kenta Nagahori, Daisuke Kiyoshima, Tsutomu Sato, Yoko Ueda, Masahito Yamamoto, Kaori Suyama, Tomoko Tanaka, Midori Yamamoto, Akifumi Eguchi, Emiko Todaka, Kenichi Sakurai, Shogo Hayashi, Haruki Yamada, Kou Sakabe

**Affiliations:** 1 Department of Environmental Preventive Medicine (Yamada Bee Company, Inc.), Center for Preventive Medical Sciences, Chiba University, 1-33 Yayoicho, Inage-ku, Chiba-si 263-8522, Chiba, Japan; kiyoshima@tokai.ac.jp (D.K.); sakabek@tokai.ac.jp (K.S.); 2 Department of Anatomy, Division of Basic Medical Science, Tokai University School of Medicine, 143 Shimokasuya, Isehara-si 259-1193, Kanagawa, Japan; nagahori.kenta.t@tokai.ac.jp (K.N.); b-sato2@tokai.ac.jp (T.S.); ueda.yoko.t@tokai.ac.jp (Y.U.); yamamoto.masahito.d@tokai.ac.jp (M.Y.); suyama@tokai.ac.jp (K.S.); sho5-884@umin.ac.jp (S.H.); 3 Laboratory of Environmental Infection Control, Louis Pasteur Center for Medical Research, 103-5 Tanaka Monzencho, Sakyo-ku, Kyoto-si 606-8225, Kyoto, Japan; 4 Department of Oral Health, The Nippon Dental University School of Life Dentistry at Tokyo, 1-9-20 Fujimi, Chiyoda-ku, Tokyo 102-8159, Japan; t-tanaka@tky.ndu.ac.jp; 5 Department of Sustainable Health Science, Center for Preventive Medical Sciences, Chiba University, 1-33 Yayoicho, Inage-ku, Chiba-si 263-8522, Chiba, Japan; midoriy@faculty.chiba-u.jp (M.Y.); a_eguchi@chiba-u.jp (A.E.); 6 Department of Global Preventive Medicine, Center for Preventive Medical Sciences, Chiba University, 1-33 Yayoicho, Inage-ku, Chiba-si 263-8522, Chiba, Japan; todakae@faculty.chiba-u.jp; 7 Department of Nutrition and Metabolic Medicine, Center for Preventive Medical Sciences, Chiba University, 1-33 Yayoicho, Inage-ku, Chiba-si 263-8522, Chiba, Japan; sakuraik@faculty.chiba-u.jp; 8 Kitasato Research Center for Environmental Science (KRCES), 1-15-1, Kitazato, Minami-ku, Sagamihara-si 252-0329, Kanagawa, Japan; yamada@kitasato-e.or.jp

**Keywords:** sulfoxaflor, mouse testis, immature, NOAEL, LOAEL

## Abstract

Sulfoxaflor, an insecticide, acts on nicotinic acetylcholine receptors. It has a functional group similar to that of neonicotinoid insecticides, which are testicular toxicants. Recently, the adverse effects of sulfoxaflor on the testes have been reported in rats. This study aimed to address the lack of reports on sulfoxaflor administration in mice and its effects on the testes. ICR mice (3- and 10-week-old) were treated ad libitum with two different concentrations (10 and 100 mg/kg) of sulfoxaflor for 4 and 8 weeks. Histological analysis and real-time reverse transcription polymerase chain reaction were performed. Testis weights relative to body weights in the sulfoxaflor groups showed no significant difference compared to the control group. Testicular tissue in the sulfoxaflor groups was unchanged compared to that in the control group. The sulfoxaflor-treated group showed no significant differences in the mRNA expression of luteinizing hormone and follicle-stimulating hormone in the pituitary gland compared to the control group. Furthermore, no significant differences were noted in the mRNA expression levels of various gene markers in the testes between the sulfoxaflor-treated and control groups. These markers include those related to Leydig cells, testosterone synthesis, Sertoli cells, proliferating cells, meiotic cells, pachytene spermatocytes, round spermatids, apoptotic cells, antioxidant enzymes, oxidative stress factors, and mitochondrial function. In contrast to findings in rats, which showed testicular toxicity, sulfoxaflor administration at low concentrations did not adversely affect intratesticular cells in either mature or immature mice at the doses and time points examined. In the future, we would like to conduct research on high concentrations of sulfoxaflor by changing the administration method.

## 1. Introduction

Agricultural chemicals have made great contributions to ensuring a stable supply of agricultural products and reducing labor input. However, toxicity to biological life, other than the target pests, and environmental pollution are critical concerns [1]. Recently, widely used neonicotinoid insecticides have been shown to strongly stimulate nicotinic acetylcholine receptors (nAChRs) [2]. Neonicotinoid insecticides cause global bee colony collapse syndrome [3,4]. Moreover, adverse health events have been reported in humans after neonicotinoid insecticide application [5]. When administered to laboratory animals, neonicotinoid insecticides adversely affect the reproductive and nervous systems owing to oxidative stress [2,5,6,7,8,9]. Neonicotinoid insecticides have adverse effects on mammals, including humans, in addition to target pests; consequently, the demand for alternative pesticides is increasing.

Sulfoxaflor was registered as a pesticide in Japan in 2017 [10]. Sulfoxaflor is used on grains (such as corn and wheat), fruit trees (such as apples and grapes), and vegetables (such as eggplant and tomatoes), with the application timing varying according to the crop type and regional regulations. Domestic shipments of sulfoxaflor-containing pesticides in Japan were approximately 5.4 t or kL in 2018 and approximately 25.5 t or kL in 2022, indicating a consistent annual increase [11]. Sulfoxaflor has functional groups in common or partially identical to those of neonicotinoid insecticides and acts as an agonist for nAChR [12,13]. Sulfoxaflor belongs to Group 4 of the IRAC (Insecticide Resistance Action Committee) mode-of-action classification, similar to neonicotinoid insecticides [14]. However, oral administration of >25 mg/kg sulfoxaflor to rats for 4 weeks adversely affects the testes due to oxidative stress [15,16,17]. Furthermore, rats are more susceptible to neonicotinoid insecticides than mice [2,8], and susceptibility to sulfoxaflor may also differ between rats and mice.

Several studies have reported that subunits (α4, β2, and α7) of nAChR are expressed in the Leydig and sperm cells of rats, mice, and humans [8,18,19,20]. Moreover, because spermatogenesis begins during puberty, the environment within the testes of adults and children is quite different [9]. Abdel-Rahman Mohamed et al. [21] reported harmful effects in the male genital system at different stages of sexual maturity in rats following imidacloprid administration. Yanai et al. also found that low doses (Non-Observed Adverse Effect Level: NOAEL) of clothianidin, a neonicotinoid insecticide, from day 1 of gestation to day 14 postpartum, decreased testis weight and the number of germ cells per seminiferous tubule in 14-day-old litter of mouse pups [22]. Furthermore, we reported that the effects of neonicotinoid insecticides on reproductive organs are different in rats and mice [2]. However, to the best of our knowledge, there is no report of the effects of sulfoxaflor on mouse reproductive organs. Therefore, in this study, we aimed to administer low concentrations of sulfoxaflor to mature and immature mice to examine its effects on the testes.

## 2. Materials and Methods

### 2.1. Animals

Male ICR mice were kept in plastic cages containing a maximum of five mice each in the Department of Laboratory Animal Science, Support Center for Medical Research and Education, Tokai University. They were maintained only in male mice. The mice were administered sulfoxaflor at 3 and 10 weeks of age. The animals were fed a standard commercial diet (CE-2; CLEA Japan, Inc., Tokyo, Japan) [23,24] and allowed to drink water during the entire experimental period. Water containing sulfoxaflor was replaced every week. Mice were maintained at a temperature of 22 ± 2 °C, 50–60% relative humidity, and a lighting period of 12 h (light/dark). No abnormal behavior was observed in any of the mice. The study was conducted according to the guidelines of the Declaration of Helsinki, and the protocols of the study were approved by the Ethics Committee of the Tokai University School of Medicine, Japan (No. 222009, 233001, 241105, 250018).

### 2.2. Dose Selection

The LD50 of Sulfoxaflor is 750 mg/kg body weight in male mice [25]. The low observable adverse effect level (LOAEL) for sulfoxaflor is 98 and 79.6 body weight/day in a 90-day and an 18-month toxicity study on mice, respectively [10,23]. Moreover, the NOAEL for sulfoxaflor is 12.8 and 10.4 mg/kg body weight/day in a 90-day and 18-month toxicity study on mice [10,23]. Additionally, the ADI of sulfoxaflor for humans is 0.042 mg/kg/day, and the ARfD is 0.25 mg/kg/day [10,23]. Furthermore, the pesticide registration retention standards established to prevent damage to aquatic organisms and plants, as well as to control water pollution, are 30 μg/L and 0.11 mg/L, respectively [10]. Therefore, two doses, 10 and 100 mg/kg, were selected for this subacute toxicity study.

### 2.3. Intake of Chemicals

Exceed flowable containing 20% sulfoxaflor (density: 1.5 g/mL) was purchased from CORTEVA agriscience Co., Ltd. (Tokyo, Japan). The different doses of excess flowable used in this study were separately dissolved in water. All mice were aged 3–10 weeks at the start of the experiments, and their diet and drinking water were freely available. This study defined administration before spermatogenesis as “immature” and administration after spermatogenesis as “mature.” The amount of sulfoxaflor was adjusted assuming that the mouse weighed 40 g and drank 6 mL/day of water daily [25].

### 2.4. Experimental Design

Mature (10-week-old) and immature (3-week-old) mice were randomly assigned to the following groups: mature control (m0, *n* = 21), immature control (i0, *n* = 12), mature 10 mg/kg (m10, *n* = 21), immature 10 mg/kg (i10, *n* = 12), mature 100 mg/kg (m100, *n* = 21), and immature 100 mg/kg (i100, *n* = 12). To avoid overstressing the experimental animals, food, sulfoxaflor-containing water, and regular water were provided ad libitum [8,9]. Because the daily water consumption per mouse was low, it was calculated by dividing the total weekly water intake by the number of mice and the number of days. The m0 and i0 groups were fed standard water for 4 and 8 weeks, respectively. The excess flowable contains 20% sulfoxaflor. However, no specific substance other than sulfoxaflor is listed in the Safety Data Sheet of the Exceed Flowable. Therefore, m0 and i0 groups were used as controls. Sulfoxaflor was administered orally via drinking water at doses of 10 and 100 mg/kg/day for 4 or 8 weeks. The 10 mg/kg/day dose was administered to mice aged 3 weeks (i10) and 8 weeks (m10), whereas the 100 mg/kg/day dose was administered to mice aged 3 weeks (i100) and 8 weeks (m100), with free access to the treated water throughout the experimental period. They were approximately the same amount as the NOAEL and LOAEL in a 90-day and 18-month toxicity mouse study [10,23]. Spermatogenesis in mice takes approximately 35 days; therefore, the exposure periods were set at 4 and 8 weeks [26]. The mice in all groups consumed approximately 5 g of diet per day. At the end of the study, at 4 and 8 weeks, the mice were anesthetized by inhalation of 3–5% isoflurane, and their body weights were recorded. Blood samples were then collected from the hearts of the mice, and serum separation was achieved through centrifugation at 3000 rpm at 4 °C for 15 min. The mice were killed by cervical dislocation. The testes and pituitary glands were removed. The testes were weighed and stored using each method.

### 2.5. Histochemistry

Mouse testes were fixed in Bouin’s Fluid for 3 days and dehydrated in 70% alcohol. Histological examination was performed using the testes from three mice per group. Testes were embedded in plastic (Technovit7100; Kulzer & Co., Wehrheim, Germany), sliced to 5 µm at 15–20 μm intervals, and placed on a glass slide. Slices were stained according to conventional methods using Gill’s hematoxylin and 2% eosin Y (Muto PC, Tokyo, Japan). Finally, the sections were observed under a light microscope.

### 2.6. Supercritical Fluid—Mass Spectrometry (SFC-MS/MS)

Sera and testes were collected from the normal and experimental groups at weeks 4 and 8, respectively. For SFC-MS/MS, three testes and four blood samples per mouse were used for each group. The sulfoxaflor concentration of the samples was outsourced to The Center of Food Safety Analysis (Miyazaki, Japan) and measured using SFC-MS/MS. Chemical standards for the sulfoxaflor analytes were obtained with a chemical purity of >95% (Hayashi Pure Chemical Ind., Ltd., Osaka, Japan). Moreover, 100 µL of mouse serum, positive control (2 nmol/µL), and negative control (sterile distilled water) were dispensed, and 800 µL of 60% acetonitrile was added. Mouse testes were homogenized in 300 µL of 60% acetonitrile (LC-MS grade, Fujifilm Wako Pure Chemical Co., Osaka, Japan), and 600 µL of 60% acetonitrile was added. After that, all samples were vortexed for 3 min, centrifuged (15,000 rpm, 4 °C, 10 min), and then dried using a Thermo Savant SPD-Series SpeedVac Concentrator (Thermo Fisher Scientific Inc., Waltham, MA, USA). These were redissolved in 1 mL of 60% acetonitrile. Vortex mixing was performed for 10 min, centrifugation was performed for 3 min (flash), the supernatant was passed through a 0.2 µm filter (Tomsic Ltd., Tokyo, Japan), and used for SFC-MS/MS. All recoveries were corrected using positive controls. Positive and negative controls were extracted using the same method for all samples.

Analysis of sulfoxaflor in prepared mouse serum and testes was performed at The Center of Food Safety Analysis (Miyazaki, Japan). The reagents used for analysis were ammonium formate (99.9% grade, Honeywell Co., Charlotte, NC, USA), chemical standard sulfoxaflor (Hayashi Pure Chemical Ind., Ltd., Osaka, Japan), methanol (LC-MS grade, Fujifilm Wako Pure Chemicals, Osaka, Japan), acetonitrile (LC-MS grade), ultrapure water (LC-MS grade) from Fujifilm Wako Pure Chemicals Corporation (Osaka, Japan), and liquefied carbon dioxide (99.9% grade, Miyazaki Oxygen Co., Miyazaki, Japan).

Analysis was performed using SFC-MS/MS (SHIMADZU Co., Kyoto, Japan) with an autosampler (SIL-30AC), carbon dioxide feed pump (LC-30AD), mobile phase feed pump (LC-20AD), BPR (SFC-30 A), make-up pump (LC-20AD), column oven (CTO-20AC), MS (LCMS8050), and LabSolutions software (version 5.98) for instrument control and data analysis.

All requested experimental samples were diluted 100-fold in 60% (*v*/*v*) acetonitrile and measured using the standard addition method. Standards were added to diluted samples at concentrations of 0, 0.5, 2, and 20 µg/L. SFC/MS analysis was performed under the following conditions. Injection volume; 1 µL, BPR pressure; 15 MPa (50 °C), column temperature; 40 °C, column; Shim-pack UC-RP (250 × 4.6 mm i.d., particle size, 5 µm, Shimadzu Corporation), make-up pump; 1 mM ammonium formate–methanol solution, flow rate 0.1 mL min^−1^, mobile phase; supercritical fluid carbon dioxide (solvent A), 0.1% (*w*/*v*) ammonium formate–methanol solution (solvent B), flow rate; 3 mL min^−1^, slope conditions; 0% B; 0 min, 0–10% B; 11 min, 10–30% B; 14 min, 30–40 B; 14.1 min, 40% B; 17 min, 40–80% B; 17.1 min, 1 mL min^−1^, 80% B; 21 min, 1 mL min^−1^, 80–100%B; 21 min, 0%B; 23.5–27 min, 3 mL min^−1^. MS analysis was performed using the following conditions: ionization method: electrospray ionization in negative mode, nebulizer flow rate: 3 L min^−1^, heating gas flow rate: 10 L min^−1^, interface temperature: 300 °C, desolvation temperature: 526 °C, DL temperature: 250 °C, heat block temperature: 400 °C. The drying gas flow rate was 10 L min^−1^. measurement mode; multiple reaction monitoring, quantitative ions; 276.30 > 212.05, qualitative ions; 276.30 > 87.95.

### 2.7. Real-Time Reverse Transcription Polymerase Chain Reaction (RT-PCR)

Pituitary glands and testes were collected from the control and experimental groups after 4 and 8 weeks of exposure. In Week 4, pituitary samples were collected from 4 mice in the m0 and m10 groups and from 5 mice in the i0, i10, i100, and m100 groups. In Week 8, pituitary samples were collected from 4 mice in the i100 and m0 groups and from 5 mice in the i0, i10, m10, and m100 groups. Testis samples collected in Week 4 included 4 samples from the m0 group and 5 samples from the i0, i10, i100, m10, and m100 groups. Similarly, in Week 8, testis samples were collected from 4 mice in the m0 group and from 5 mice in the i0, i10, i100, m10, and m100 groups. Real-time RT-PCR was performed using both the pituitary gland and the testicular tissues. Total RNA was isolated from each group’s testes and pituitary gland using the TRIzol RNA extraction and reverse-transcribed into cDNA using the Kit (High-Capacity cDNA Reverse Transcription Kit, Thermo Fisher Scientific Inc., Waltham, MA, USA) according to the manufacturer’s instructions. Quantification of cDNA was performed using a Cobas Z 480 Analyzer (Roche Diagnostics K.K., Tokyo, Japan). The sequences (Table 1) of Steroidogenic acute regulatory protein (Star), choleserol side-chain cleavage cytochrome P450 (P450scc), 17 α-hydoroxylase/17, 20 lyase (P450c17), 3β-Hydroxysteroid dehydrogenase (3β-HSD), 17β-Hydroxysteroid dehydrogenase (17-HSD), luteinizing hormone receptor (LHR), follicle-stimulating hormone receptor (FSHR), Inhibin A (InhA), sex hormone-binding globulin (Shbg), topoisomerase 2-alpha (Top2a), synaptonemal complex protein 3 (Sycp3), MutL homolog 1 (Mlh1), Acrosin (Acr), transition protein 1 (Tnp), Fas, Fas ligand (Fasl), Bax, Bcl-2, Caspase3, glutathione peroxidase (GPx), catalase (CAT), superoxide dismutase 1 (SOD1), SOD2, thioredoxin 1 (Trx1), Trx2, thioredoxin-interacting protein (TXNIP), nuclear factor E2-related factor 2 (Nrf2), 8-oxoguanine DNA glycosylase 1 (OGG1), adrenoleukodystrophy protein belonging to sub-family D, member 1 (ABCD1), ABCD3, glucose-regulated protein, 78kDa (GRP78), peroxisome proliferators-activated receptor-γ co-activator-1α (PGC-1), Nrf1, mitochondrial transcription factor A (Tfam), dynamin-related protein 1 (Drp1), mitochondrial fission 1 protein (Fis1), Mitofusin 1 (Mfn1), optic atrophy protein 1 (Opa1), luteinizing hormone (LH), follicle-stimulating hormone (FSH) were obtained from FASMAC. The data were analyzed using the Light Cycler® 480SW-User Defined Workflow for Cobas Z 480 Analyzer (Roche Diagnostics K.K., Tokyo, Japan), and the comparative Ct method (2^−ΔΔCt^) was used to quantify gene expression levels. Real-time RT-PCR data were standardized to the internal control GAPDH.

### 2.8. Statistical Analysis

Data are expressed as mean ± standard deviation (SD). IBM SPSS 31 (International Business Machines Co., Armonk, NY, USA) statistics were used to analyze the data. This study was analyzed using analysis of variance, Kruskal–Wallis test, and multiple comparisons (Tukey HSD test and Dunn–Bonferroni test) based on the results of the Shapiro–Wilk test. Differences were considered statistically significant at *p* < 0.05. It was determined that there was no significant difference between the results of real-time PCR unless the values were doubled or more than half, even if there was a statistically significant difference [53].

## 3. Results

### 3.1. Physical Data of Mice in 4 and 8 Weeks After Treatment with Sulfoxaflor

**Figure 1:**
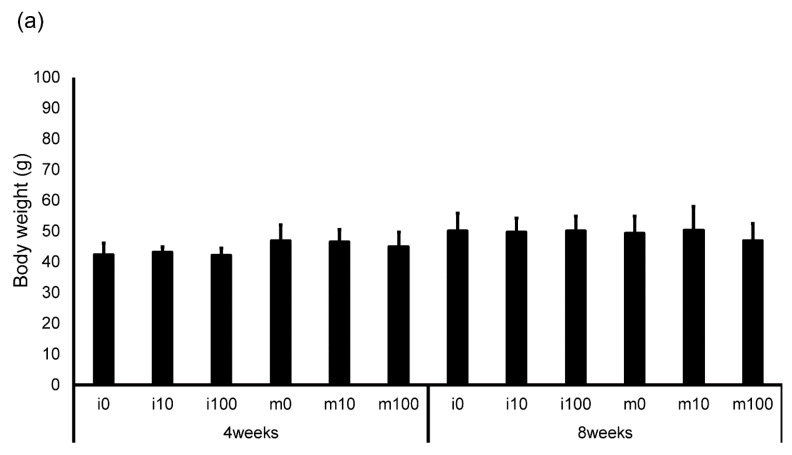

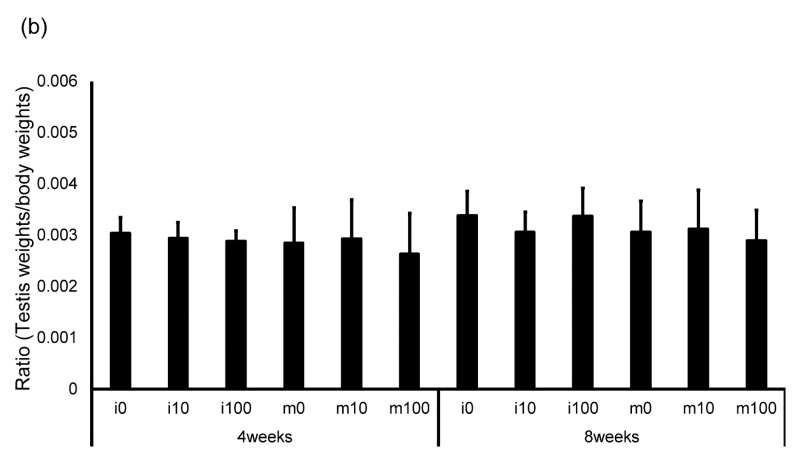
Changes in body weight (**a**) and testis weight relative to body weight (**b**) in control and sulfoxaflor-treated mice at the immature and mature stages. Mice aged 3 weeks (i0, i10, i100) and 8 weeks (m0, m10, m100) were exposed to normal water (i0 and m0), sulfoxaflor at 10 mg/kg (i10 and m10), or sulfoxaflor at 100 mg/kg (i100 and m100) for 4 or 8 weeks. The mean values are plotted. Bars, standard deviation of the mean.

The average amount of water consumed by the mice and the average amount of sulfoxaflor administered to them are shown in Table 2. The drinking water for the i0 and m0 groups, which were not administered sulfoxaflor, is indicated as “–” because it did not contain the compound. The average sulfoxaflor concentration ranged from 9.9 to 13.3 mg/kg for the i10 and m10 groups (planned dose: 10 mg/kg) and from 81.2 to 118.1 mg/kg for the i100 and m100 groups (planned dose: 100 mg/kg). Body weights 4 weeks after administration of normal water or sulfoxaflor were approximately 42.5 g (i0), 43.1 g (i10), 42.2 g (i100), 47.0 g (m0), 46.6 g (m10), and 44.9 g (m100). After 8 weeks, body weights were approximately 50.1 g (i0), 49.7 g (i10), 50.2 g (i100), 49.4 g (m0), 50.3 g (m10), and 46.9 g (m100). The testicular weight relative to body weight 4 weeks after administration was approximately 0.003 (i0), 0.0029 (i10), 0.0029 (i100), 0.0029 (m0), 0.0029 (m10), and 0.0026 (m100). After 8 weeks, the corresponding ratios were approximately 0.003 (i0), 0.003 (i10), 0.003 (i100), 0.0031 (m0), 0.0031 (m10), and 0.0029 (m100). The body weight of immature mice after sulfoxaflor administration showed no significant differences between groups at either 4 weeks (*p* = 0.887, η^2^ = 0.017) or 8 weeks (*p* = 0.987, η^2^ = 0.002) (Figure 1a). Similarly, in mature mice, no significant differences were observed at 4 weeks (*p* = 0.580, η^2^ = 0.041) or 8 weeks (*p* = 0.489, η^2^ = 0.040) (Figure 1a). Testis weight relative to body weight also showed no significant differences between the groups. In immature mice, the *p*-values were 0.974 (η^2^ = 0.004) at 4 weeks and 0.503 (η^2^ = 0.108) at 8 weeks (Figure 1b). In mature mice, the *p*-values were 0.517 (r = 0.217) at 4 weeks and 0.780 (η^2^ = 0.014) at 8 weeks (Figure 1b).

### 3.2. Histochemical Observation

**Figure 2:**
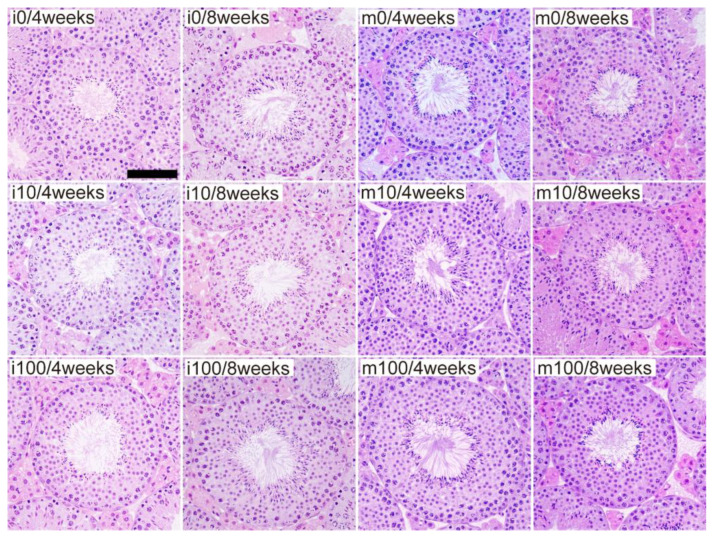
High-magnification testicular histology of control and sulfoxaflor-treated mice at immature and mature stages. Mice aged 3 weeks (i0, i10, i100) and 8 weeks (m0, m10, m100) were exposed to normal water (i0 and m0), sulfoxaflor at 10 mg/kg (i10 and m10), or sulfoxaflor at 100 mg/kg (i100 and m100) for 4 or 8 weeks. Scale bar: 100 µm.

Compared with the control groups (i0 and m0), no morphological changes were observed in the low-magnification images of testicular tissue from mice in the sulfoxaflor-treated groups (i10, m10, i100, and m100) (Figure S1). High-magnification observations of individual seminiferous tubules in the testicular tissue shown in Figure S1 also revealed no morphological alterations in the i10, m10, i100, or m100 groups compared to the i0 and m0 groups (Figure 2). Similarly, low-magnification observations of testicular tissue from other mice showed no morphological differences between the sulfoxaflor-treated (i10, m10, i100, and m100) and control (i0 and m0) groups (Figure S2).

### 3.3. Average Sulfoxaflor Intake and Average Content of Sulfoxaflor in the Serum and Testis

The concentration of sulfoxaflor in serum was approximately 1.4 and 1.5 µg/mL in the i10 group; 1.6 and 1.5 µg/mL in the m10 group; 12.7 and 14.2 µg/mL in the i100 group; and 14.0 and 10.2 µg/mL in the m100 group at 4 and 8 weeks post-administration, respectively (Table 2). The concentration of sulfoxaflor per gram of testis was approximately 1.3 and 1.3 µg/g in the i10 group; 1.5 and 1.2 µg/g in the m10 group; 10.4 and 9.0 µg/g in the i100 group; and 12.3 and 9.3 µg/g in the m100 group at 4 and 8 weeks post-administration, respectively (Table 2). Sulfoxaflor concentrations in both the serum and testes increased proportionally with the administered dose (Table 2).

### 3.4. Effects of Sulfoxaflor on the Testis and Pituitary mRNA

**Figure 3:**
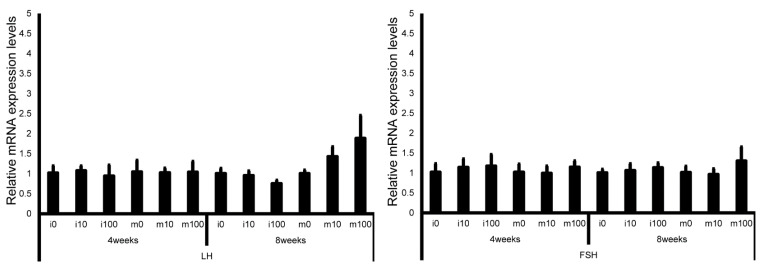
mRNA expression levels of luteinizing hormone (LH) and follicle-stimulating hormone (FSH) in the pituitary glands of control and sulfoxaflor-treated mice at the immature and mature stages. β-Actin expression was used to normalize target gene expression levels. Mice aged 3 weeks (i0, i10, i100) and 8 weeks (m0, m10, m100) were exposed to normal water (i0 and m0), sulfoxaflor at 10 mg/kg (i10 and m10), or sulfoxaflor at 100 mg/kg (i100 and m100) for 4 or 8 weeks. Thick and thin bars represent mean values and standard deviation of the mean, respectively.

Pituitary gonadotropin marker (LH, FSH) mRNA levels in immature and mature mice at 4 and 8 weeks after sulfoxaflor administration were not significantly different among all groups (Figure 3).

**Figure 4:**
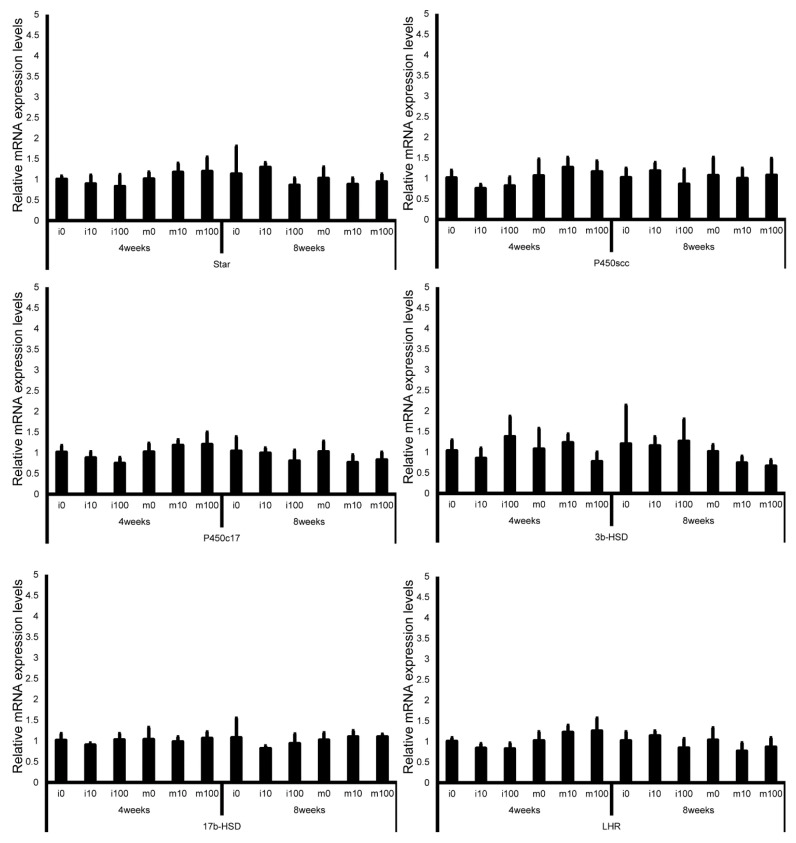
mRNA expression levels of Leydig cell–associated genes and testosterone synthesis–related genes (steroidogenic acute regulatory protein (Star), cholesterol side-chain cleavage cytochrome P450 (P450scc), 17α-hydroxylase/17,20-lyase (P450c17), 3β-hydroxysteroid dehydrogenase (3β-HSD), 17β-hydroxysteroid dehydrogenase (17β-HSD), and luteinizing hormone receptor (LHR)) in the testes of control and sulfoxaflor-treated mice at immature and mature stages. Mice aged 3 weeks (i0, i10, i100) and 8 weeks (m0, m10, m100) were exposed to normal water (i0 and m0), sulfoxaflor at 10 mg/kg (i10 and m10), or sulfoxaflor at 100 mg/kg (i100 and m100) for 4 or 8 weeks. β-actin expression was used for the normalization of the target gene expression levels. Thick and thin bars represent mean values and standard deviation of the mean, respectively.

**Figure 5:**
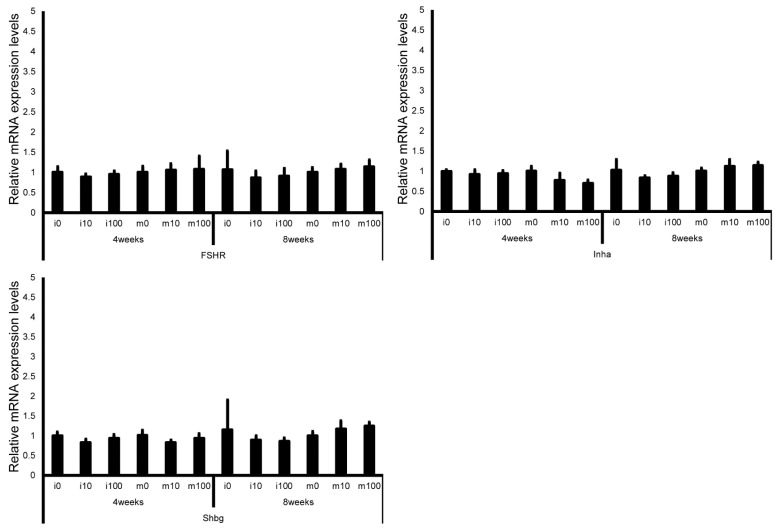
mRNA expression levels of Sertoli cell–associated genes (follicle-stimulating hormone receptor (FSHR), inhibin A (InhA), and sex hormone-binding globulin (Shbg)) in the testes of control and sulfoxaflor-treated mice at immature and mature stages. Mice aged 3 weeks (i0, i10, i100) and 8 weeks (m0, m10, m100) were exposed to normal water (i0 and m0), sulfoxaflor at 10 mg/kg (i10 and m10), or sulfoxaflor at 100 mg/kg (i100 and m100) for 4 or 8 weeks. β-actin expression was used for the normalization of the target gene expression levels. Thick and thin bars represent the mean values and standard deviation of the mean, respectively.

**Figure 6:**
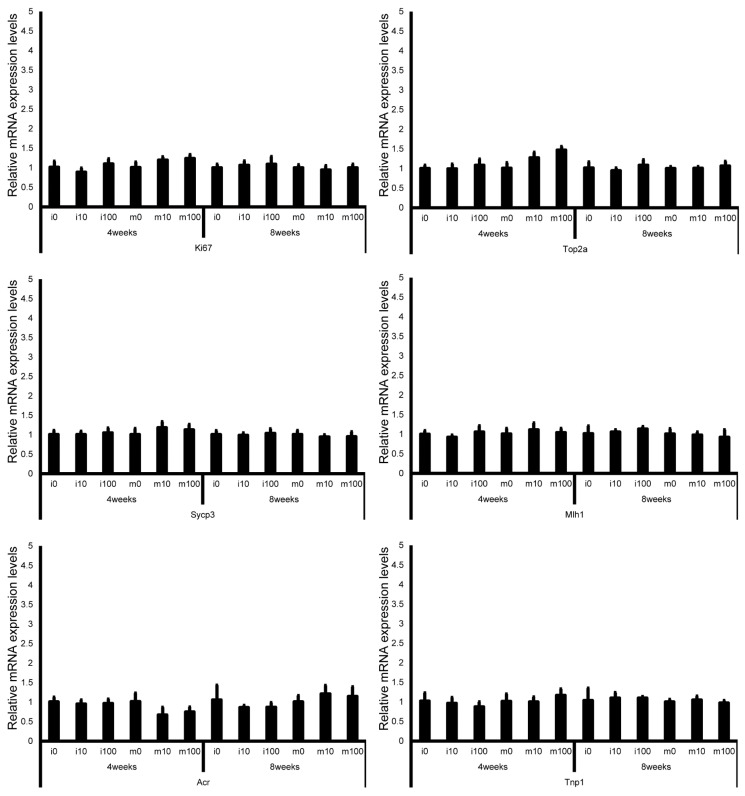
mRNA expression levels of germ cell–associated genes (Ki67, topoisomerase 2-alpha (Top2a), synaptonemal complex protein 3 (Sycp3), MutL homolog 1 (Mlh1), acrosin (Acr), and transition protein 1 (Tnp1)) in the testes of control and sulfoxaflor-treated mice at immature and mature stages. Mice aged 3 weeks (i0, i10, i100) and 8 weeks (m0, m10, m100) were exposed to normal water (i0 and m0), sulfoxaflor at 10 mg/kg (i10 and m10), or sulfoxaflor at 100 mg/kg (i100 and m100) for 4 or 8 weeks. β-actin expression was used for the normalization of the target gene expression levels. Thick and thin bars represent the mean values and standard deviation of the mean, respectively.

**Figure 7:**
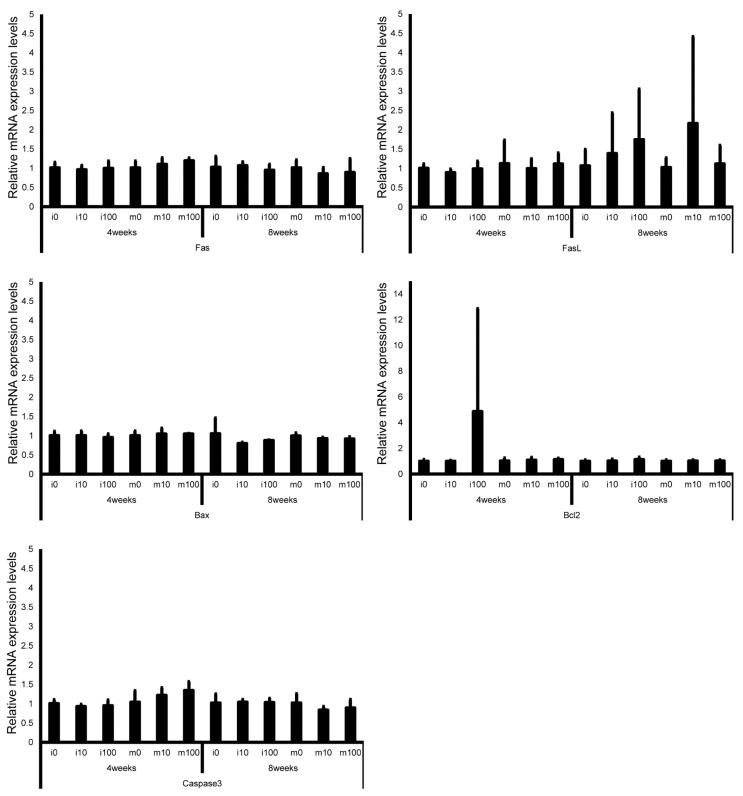
mRNA expression levels of apoptosis-related genes (Fas, Fas ligand, Bax, Bcl-2, and Caspase3) in the testes of control and sulfoxaflor-treated mice at the immature and mature stages. Mice aged 3 weeks (i0, i10, i100) and 8 weeks (m0, m10, m100) were exposed to normal water (i0 and m0), sulfoxaflor at 10 mg/kg (i10 and m10), or sulfoxaflor at 100 mg/kg (i100 and m100) for 4 or 8 weeks. β-actin expression was used for the normalization of the target gene expression levels. Thick and thin bars represent the mean values and standard deviation of the mean, respectively.

**Figure 8:**
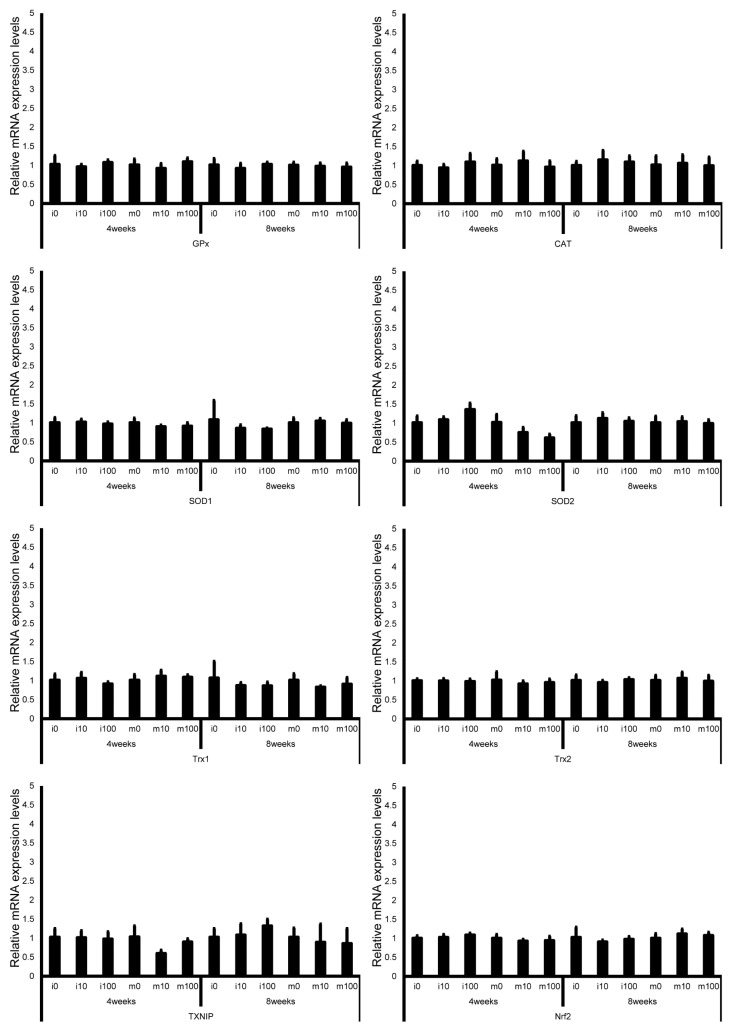
mRNA expression levels of antioxidant enzyme–associated genes (glutathione peroxidase (GPx), catalase (CAT), superoxide dismutase 1 (SOD1), thioredoxin 1 (Trx1), thioredoxin 2 (Trx2), thioredoxin-interacting protein (TXNIP), and nuclear factor erythroid 2–related factor 2 (Nrf2)) in the testes of control and sulfoxaflor-treated mice at immature and mature stages. Mice aged 3 weeks (i0, i10, i100) and 8 weeks (m0, m10, m100) were exposed to normal water (i0 and m0), sulfoxaflor at 10 mg/kg (i10 and m10), or sulfoxaflor at 100 mg/kg (i100 and m100) for 4 or 8 weeks. β-actin expression was used for the normalization of the target gene expression levels. Thick and thin bars represent the mean values and standard deviation of the mean, respectively.

**Figure 9:**
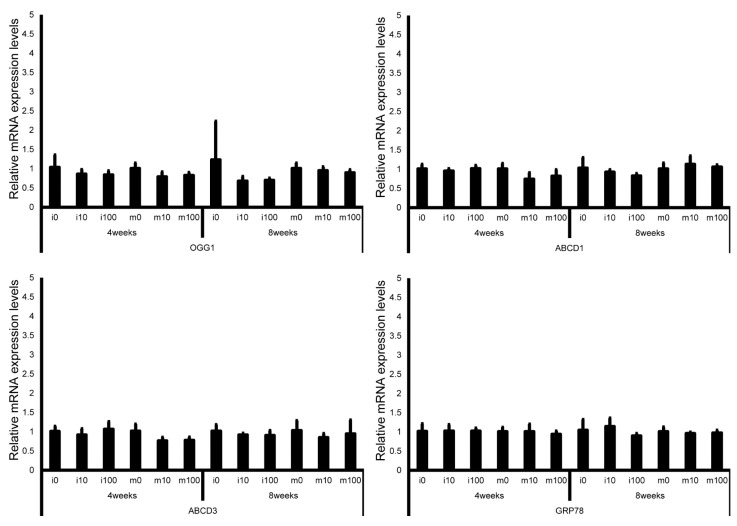
mRNA expression levels of oxidative stress–related genes (8-oxoguanine DNA glycosylase 1 (OGG1), adrenoleukodystrophy protein sub-family D member 1 (ABCD1), ATP-binding cassette sub-family D member 3 (ABCD3), and glucose-regulated protein 78 kDa (GRP78)) in the testes of control and sulfoxaflor-treated mice at immature and mature stages. Mice aged 3 weeks (i0, i10, i100) and 8 weeks (m0, m10, m100) were exposed to normal water (i0 and m0), sulfoxaflor at 10 mg/kg (i10 and m10), or sulfoxaflor at 100 mg/kg (i100 and m100) for 4 or 8 weeks. β-actin expression was used for the normalization of the target gene expression levels. Thick and thin bars represent the mean values and standard deviation of the mean, respectively.

**Figure 10:**
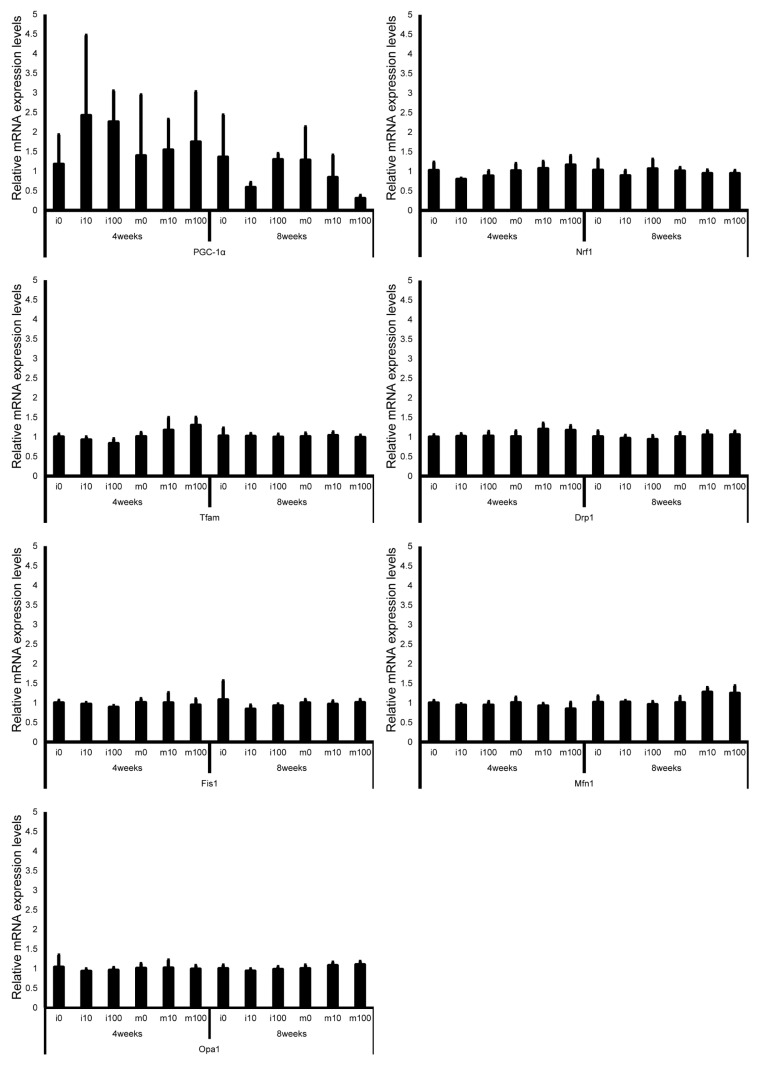
mRNA expression levels of mitochondrial factor–associated genes (peroxisome proliferator–activated receptor γ co-activator 1α (PGC-1α), nuclear respiratory factor 1 (Nrf1), mitochondrial transcription factor A (Tfam), dynamin-related protein 1 (Drp1), mitochondrial fission 1 protein (Fis1), mitofusin 1 (Mfn1), and optic atrophy protein 1 (Opa1)) in the testes of control and sulfoxaflor-treated mice at immature and mature stages. Mice aged 3 weeks (i0, i10, i100) and 8 weeks (m0, m10, m100) were exposed to normal water (i0 and m0), sulfoxaflor at 10 mg/kg (i10 and m10), or sulfoxaflor at 100 mg/kg (i100 and m100) for 4 or 8 weeks. Thick and thin bars represent the mean values and standard deviation of the mean, respectively.

Leydig cell and testosterone synthesis markers (Star, P450scc, P450c17, 3β-HSD, 17β-HSD, and LHR; Figure 4), Sertoli cell markers (FSHR, InhA, and Shbg; Figure 5), proliferating cell marker (Ki67; Figure 6), meiotic cell marker (Top2a; Figure 7), pachytene spermatocyte markers (Sycp3 and Mlh1; Figure 6), round spermatid markers (Acr and Tnp1; Figure 6), apoptotic cell markers (Fas, FasL, Bax, Bcl-2, and Caspase3; Figure 7), antioxidant enzyme markers (GPx, CAT, SOD1, Trx1, Trx2, TXNIP, and Nrf2; Figure 8), oxidative factor marker (OGG1, ABCD1, ABCD3, and GRP78; Figure 9), mitochondrial factor markers (PGC-1α, Nrf1, Tfam, Drp1, Fis1, Mfn1, and Opa1; Figure 10) mRNA levels in immature and mature mice at 4 and 8 weeks after sulfoxaflor administration were not significantly different among all groups. The *p*-values and effect sizes (η^2^ or r) for all gene comparisons are shown in Table 3.

## 4. Discussion

To the best of our knowledge, this is the first study to report the effects of sulfoxaflor treatment on mature and immature mice. This study demonstrated that exposure to sulfoxaflor in drinking water (NOAEL and LOAEL concentrations) for 4 and 8 weeks did not change body weight, testicular weight relative to body weight, or testicular histology of mature and immature mice compared to the normal group. The concentration of sulfoxaflor in the serum and testes was 1/10 of the intake level. No significant difference was observed in the mRNA levels of pituitary gonadotropin Leydig cell, testosterone synthesis, Sertoli cell, germ cell, apoptotic cell, antioxidant enzyme, oxidative factor, and mitochondrial factor markers compared with the normal control group.

In 7-week-old male rats, oral exposure to sulfoxaflor at 100 mg/kg for 4 weeks resulted in its higher accumulation in the liver and kidneys than in other organs, causing mild inflammation in the liver and kidneys [54]. Additionally, in male rats, oral exposure to sulfoxaflor at 24.8 and 79.4 mg/kg/day for 4 weeks resulted in increased white blood cell counts, serum levels of cytokines (interleukin (IL)-1β, tissue necrosis factor-α, interferon-γ, IL-4), malondialdehyde in the spleen and thymus, increased CD3 and CD11b gene expression, decreased red blood cell count, hemoglobin content, serum total antioxidant capacity, catalase, and superoxide dismutase in the spleen and thymus, and caused inflammation in the spleen and thymus tissue, leading to oxidative stress-induced inflammation of the lymphoid organs [55].

In male mice aged 32–40 weeks, blood tests conducted after oral exposure to sulfoxaflor at 15 mg/kg/day for 1 and 7 days revealed elevated levels of 8-hydroxy-2′-deoxyguanosine, carbonylated proteins, glutathione, aspartate aminotransferase (after 1 d) activity, lactate dehydrogenase (after 1 d) activity, malondialdehyde (after 7 days), hemoglobin concentration (after 7 days), and a decrease in white blood cell count and mean corpuscular hemoglobin concentration [56]. Additionally, in mature male mice, brain examination results following oral exposure to sulfoxaflor at 15 mg/kg/day for 1 and 7 days showed increased caspase-3 gene expression, lipid peroxidation levels, glutathione reductase-specific enzyme activity, glutathione transferase (after 7 days), glutathione peroxidase (after 7 days) activity, and a decrease in total glutathione (after 7 days), indicating that oxidative stress in the brain induced apoptosis [57]. Additionally, experiments involving the administration of sulfoxaflor to adult rodents have reported testicular [15,16,17] and developmental toxicity [58,59]. Thus, the administration of sulfoxaflor to rats and mice suggests that oxidative stress may cause damage to the spleen, thymus, brain, and blood.

However, when sulfoxaflor was orally administered to male and female mice at doses of 750 mg/kg and 1250 mg/kg, respectively, an increase in hepatocellular adenomas and hepatocellular carcinomas was observed after 18 months. In contrast, when sulfoxaflor was orally administered to male rats at a dose of 500 mg/kg, an increase in hepatocellular adenomas was observed after 2 years [60]. Sensitivity to sulfoxaflor differed between rats and mice. Additionally, when sulfoxaflor was orally administered at approximately 26.4 mg/kg/day to rats after mating, it caused fetal defects (forelimb flexion, hindlimb rotation, and clavicle flexion) and neonatal mortality. However, in a dietary study in rabbits, despite achieving similar maternal and fetal plasma exposure levels, there were no effects on the fetus or neonates. Thus, because sensitivity varies greatly among experimental animals, animal experiments involving various species are necessary.

Mohamed et al. orally treated 10–12-week-old rats with 25 and 100 mg/kg sulfoxaflor for 4 weeks and reported increased levels of LH (100 mg/kg), malondialdehyde (25 and 100 mg/kg), and glutathione peroxidase (100 mg/kg), as did the percentage of dead (25 and 100 mg/kg), abnormal (25 and 100 mg/kg), and DNA-damaged sperm cells (100 mg/kg) [17]. Said et al. orally treated mature rats with 79.5 mg/kg/day of sulfoxaflor for 4 weeks and reported increased malondialdehyde and nitrogen oxides levels, caspase3 activity, and decreased spermatogenic capacity and cellular energy parameters [16]. Furthermore, Tijani et al. orally treated mature rats with 79.4 mg/kg/day of sulfoxaflor for 4 weeks and reported increased relative testicular weights, abnormal sperm morphology percentage, testicular levels of tumor necrosis-α, interleukin-1β, nitric oxide, serum LH concentration, serum FSH concentration, testicular activities of xanthine oxidase, myeloperoxidase, protein oxidation level, reactive oxygen and nitrogen species level, myeloperoxidase, and caspase3, and decreased testicular activities of acid phosphatase, alkaline phosphatase, glucose-6-phosphate dehydrogenase, lactate dehydrogenase, SOD, CAT, GPx, reduced glutathione and total antioxidant capacity, serum testosterone concentration, and testicular levels of interleukin-10 [15]. Thus, sulfoxaflor adversely affects the testes of rats due to oxidative stress. However, a 4-week administration of sulfoxaflor to mice had little effect on the mRNA levels of pituitary gonadotropin, Leydig cells, testosterone synthesis, Sertoli cells, germ cells, apoptotic cells, antioxidant enzymes, oxidative factors, and mitochondrial factor markers in this study. Sulfoxaflor administered for 8 weeks showed similar results, indicating that sulfoxaflor is less toxic in mice than in rats. In this study, no differences were observed in the mRNA expression or histological features of the testes between the control and treatment groups; therefore, analyses of proteins or their functions were not performed. The non-toxic dose of sulfoxaflor in a 90-day subacute toxicity study was 6.36 mg/kg/day in rats, despite a dose of 12.8 mg/kg/day in mice [10]. In addition, the concentration of imidacloprid, a neonicotinoid pesticide that lowers serum testosterone concentration, was 30 mg/kg/day in mice [61] and 1 mg/kg/day in rats [21]. The difference between the digestive tracts of mice and rats is that mice possess a gallbladder, whereas rats do not. This distinction affects the absorption processes in the digestive tract, particularly lipid absorption. In mammals, neonicotinoid insecticides are primarily metabolized by hepatic microsomal cytochrome P450 (CYP) enzymes and cytoplasmic aldehyde oxidase [55]. Metabolism via CYP2A6, CYP2B6, CYP2C19, and CYP3A4 has been confirmed [62], and the resulting metabolites can sometimes exhibit strong toxicity [5,55]. Studies investigating species differences in CYP-mediated drug metabolism in mice, rats, dogs, monkeys, and humans have revealed that species-specific isoforms of CYP1A, CYP2C, CYP2D, and CYP3A cause significant variations in catalytic activity [63]. Experiments using liver microsomal fractions from rats, dogs, cats, and humans with the neonicotinoid insecticides imidacloprid and acetamiprid have also demonstrated substantial interspecies differences in metabolite formation rates [64]. Thus, neonicotinoid insecticide metabolism varies among species. To the best of our knowledge, no studies have directly compared metabolites or testicular toxicity in mice and rats; however, differences in metabolism mediated by CYP enzymes or aldehyde oxidase may exist. In mice, metabolism of the neonicotinoid insecticide thiamethoxam efficiently generates more toxic metabolites, resulting in hepatotoxicity and hepatocarcinogenicity in mice, but not in rats [65]. Furthermore, although testicular toxicity has been reported in rats, no such effects have been documented in mice. These findings suggest that organ sensitivity, including that of the testes, differs among species [64]. Therefore, compared to rats, mice may exhibit lower testicular sensitivity to sulfoxaflor and its metabolites.

Spermatozoa did not appear in the testicular epithelium until puberty, indicating differences in the environment within the testes between adults and children. Bal et al. [66,67] administered imidacloprid at doses of 0.5, 2, and 8 mg/kg to immature rats (1 week old) and mature rats (8–9 weeks old) for 90 days. The results showed that immature rats exhibited testicular damage at low concentrations, whereas mature rats only exhibited testicular damage at high concentrations [66,67]. A similar experiment was conducted using clothianidin (2, 8, and 24 mg/kg). While mature rats (8–9 weeks old) showed little harmful effect on the reproductive system, immature rats (1 week old) exhibited adverse effects on the testes and epididymis [68,69]. However, Abdel-Rahman Mohamed et al. [21] reported that when 1 mg/kg of imidacloprid was administered to immature rats (4 weeks old) and mature rats (7 weeks old) for 65 days, both groups exhibited adverse effects on the testes, with the effects being more pronounced in mature rats than in immature rats. Thus, the testicular toxicity of neonicotinoids in rats may vary in severity depending on the maturity stage (week of age). In this study, there were no differences in the effects of 10 or 100 mg/kg sulfoxaflor between the immature and mature mice. Increasing the sulfoxaflor dose may reveal differences between immature and mature mice.

Acetamiprid accumulation levels were 0.3 pg/μL and 100 pg/mg in the blood and testes, respectively, of mature rats treated with 3.5 mg/kg (approximately 350 g)/day [70], and 1.8 pg/μL and 900 pg/mg in the blood and testes, respectively, in immature rats treated with 10.5 mg/kg (approximately 350 g)/day of acetamiprid [70]. Contrary to this, acetamiprid concentrations of 2.6 mg/day reached approximately 0.4 pg/μL and 0.3 pg/mg in the blood and testes of mice, respectively. In comparison, 23.4 mg/day reached approximately 63.9 pg/μL and 7.1 pg/mg, respectively [8]. Despite the high blood concentrations of acetamiprid, it is less likely to accumulate in the mouse testes than in the rat testes. The blood and testicular concentrations of sulfoxaflor in mice were determined in this study. However, such reports in rats are lacking, which we hope will be evaluated in future studies.

## 5. Conclusions

This study demonstrated that exposure to sulfoxaflor (10 and 100 mg/kg) for 4 or 8 weeks caused no changes in the testes of mice at the doses and time points examined. In contrast, administration of sulfoxaflor at 25–100 mg/kg for 4 weeks in rats has been reported to induce testicular toxicity through oxidative stress, whereas administration to both mature and immature mice did not produce harmful effects on testicular cells. However, a limitation of this study is that because no reports are available on sulfoxaflor concentrations in the blood or testes of rats, a direct comparison could not be made. In addition, this study was conducted under conditions of ad libitum water intake, and since higher concentrations reached the humane endpoint in preliminary experiments, sulfoxaflor administration was restricted to lower concentrations. In the future, we would like to conduct research on high concentrations of sulfoxaflor using a sonde or catheter.

## Figures and Tables

**Table 1:** Primer sequences.

Marker	Name	Forward Primer (5′-3′)	Reverse Primer (5′-3′)	Reference
Pituitary gland	LH	CTGAGCCCAAGTGTGGTGT	CACAGATGCTGGTGGTGAAG	[27]
	FSH	AAGTC ATCCAGCTTTGCAT	TCCCTGGTGTAGCAGTAGCC	[27]
Leydig cell	Star	GCATACTCAACAACCAGGAAGG	CTGGTTGATGATTGTCTTCGGC	[28]
	P450scc	AGGTCCTTCAATGAGATCCCTT	TCCCTGTAAATGGGGCCATAC	[29]
	P450c17	GATCGGTTTATGCCTGAGCG	TCCGAAGGGCAAATAACTGG	[30]
	3βHSD	CCTCCGCCTTGATACCAGC	TTGTTTCCAATCTCCCTGTGC	[29]
	17βHSD	ACTTGGCTGTTCGCCTAGC	GAGGGCATCCTTGAGTCCTG	[29]
	LHR	CCTTGTTCTCAAAAGAGATGTTGAA	TGACCAGAAACTGGAGAAGATGATA	[31]
Sertoli cell	FSHR	ATTTTCTCCAGGTCCCCAAA	TCTCCTTGCTGGCATTCTTG	[31]
	Inha	CTCGAAGACATGCCGTTGG	AGCT GGCTGGTCCTCACAG	[32]
	Shbg	GCC CTG AGA CAC ATT GAC CCT	CAG GGC AGG CAG GAG CG	[33]
Germ cell	Ki67	CCATATGCCTGTGGAGTGGAA	CCACCCTTAGCGTGCTCTTGA	[34]
	Top2A	CAACTGGAACATATACTGCTCCG	GGGTCCCTTTGTTTGTTATCAGC	[35]
Pachytene spermatocyte	Sycp3	TCAGCAGAGAGCTTGGTCGG	GATGTTTGCTCAGCGGCTCC	[36]
	Mlh1	TTCTACCTATGGCTTTCGTGGTGA	TGCCTGCACAGGGTTTAGGAG	[37]
Round spermatids	Acr	TCCTGAAGGCAAGATTGACACC	TAGACTCCGGGACGCTTAGCAC	[38]
	Tnp1	ACCAGCCGCAAGCTAAAGAC	TTTCCTACTTTTCAGGACGCTC	[39]
Apoptosis	Fas	CAGACATGCTGTGGATCTGG	CACAGTGTTCACAGCCAGGA	[40]
	Fas ligand	GCTCTTCCACCTGCAGAAG	ATTCCTCAAAATTGATCAGAGAGAG	[40]
	Bax	CACCAAGAAGCTGAGCGAGT	GCCCCAGTTGAAGTTGCCAT	[41]
	Bcl-2	CTGAGTACCTGAACCGGCATC	AGAAATCAAACAGAGGTCGCAT	[41]
	Caspase3	AAAGGCTGGAACCCTTGTTT	GCACCTTGCCTTCAATGAGT	[42]
Antioxidant	GPx	TTGAGAAAGGAGATGTGAACGG	CAAAGTTCCAGCGGATGTCA	[42]
	CAT	CTCAGGTGCGGACATTCTATAC	GACTCCATCCAGCGATGATTAC	[43]
	SOD1	GCTGTACCAGTGCAGGACCTCAT	CTCTCCTGAGAGTGAGATCACACGA	[44]
	SOD2	ATGGTGGGGGACATATT	GAACCTTGGACTCCCACAGA	[44]
	Trx1	TCAAGCCCTTCTTCCATTCC	GTCGGCATGCATTTGACTTC	[45]
	Trx2	CGCGGCTAGAGAAGATGGTC	TTGATGGCTAGCACGGTAGG	[45]
	TXNIP	AAGCTGTCCTCAGTCAGAGGCAAT	ATGACTTTCTTGGAGCCAGGGACA	[45]
	Nrf2	TCTCCTAGTTCTCCGCTGCT	GTTTGGGAATGTGGGCAACC	[46]
Oxidizing substances	OGG1	GATTGGACAGTGCCGTAA	GGAAGTGGGAGTCTACAG	[47]
	ABCD1	GAGGGAGGTTGGGAGGCAGT	GAGGGAGGTTGGGAGGCAGT	[48]
	ABCD3	CTGGGCGTGAAATGACTAGATTGG	AGCTGCACATTGTCCAAGTACTCC	[48]
	GRP78	AACCCAGATGAGGCTGTAGCA	ACATCAAGCAGAACCAGGTCAC	[49]
Mitochondria damage	PGC-1α	AGGAAATCCGAGCGGAGCTGA	GCAAGAAGGCGACACATCGAA	[50]
	Nrf1	AGCACGGAGTGACCCAAAC	TGTACGTGGCTACATGGACCT	[51]
	Tfam	ATTCCGAAGTGTTTTTCCAGCA	TCTGAAAGTTTTGCATCTGGGT	[51]
	Drp1	ATGCCAGCAAGTCCACAGAA	TGTTCTCGGGCAGACAGTTT	[52]
	Fis1	CAAAGAGGAACAGCGGGACT	ACAGCCCTCGCACATACTTT	[52]
	Mfn1	GCAGACAGCACATGGAGAGA	GATCCGATTCCGAGCTTCCG	[52]
	Opa1	ACCTTGCCAGTTTAGCTCCC	TTGGGACCTGCAGTGAAGAA	[52]
House keeping	GAPDH	TGAACGGGAAGCTCACTGG	TCCACCACCCTGTTGCTGTA	[40]

**Table 2:** Average intake in control and sulfoxaflor-treated mice at immature and mature stages and average sulfoxaflor concentrations in the testes and serum. Mice aged 3 weeks (i0, i10, i100) and 8 weeks (m0, m10, m100) were exposed to normal water (i0 and m0), sulfoxaflor at 10 mg/kg (i10 and m10), or sulfoxaflor at 100 mg/kg (i100 and m100) for 4 or 8 weeks. Sulfoxaflor concentrations are presented as mean values ± standard deviation.

		Drink (SD)	Intake (SD)	Serum (SD)	Testis (SD)
Term	Group	(mL/day)	(μg/g/day)	(μg/mL)	(μg/g)
4 weeks	i0	7.9 (1.4)	-	N.D.	N.D.
	i10	8.2 (1.3)	13.3 (2.0)	1.4 (0.5)	1.3 (0.7)
	i100	7.2 (1.1)	118.1 (18.8)	12.7 (0.8)	10.4 (2.4)
	m0	8.2 (1.8)	-	N.D.	N.D.
	m10	8.3 (1.4)	12.5 (2.1)	1.56 (0.4)	1.47 (0.7)
	m100	6.2 (1.1)	98.2 (16.6)	14.04 (2.4)	12.33 (2.8)
8 weeks	i0	7.5 (1.3)	-	N.D.	N.D.
	i10	7.1 (1.3)	9.9 (1.8)	1.5 (0.4)	1.3 (0.2)
	i100	7.0 (1.0)	96.2 (13.8)	14.2 (4.3)	9.0 (4.6)
	m0	7.4 (1.1)	-	N.D.	N.D.
	m10	8.0 (1.1)	10.8 (1.4)	1.51 (0.6)	1.2 (0.4)
	m100	5.6 (0.9)	81.2 (13.2)	10.2 (3.0)	9.28 (0.9)

**Table 3:** Statistical results for each gene on the testis and pituitary mRNA. Mice aged 3 weeks (i0, i10, i100) and 8 weeks (m0, m10, m100) were exposed to normal water (i0 and m0), sulfoxaflor at 10 mg/kg (i10 and m10), or sulfoxaflor at 100 mg/kg (i100 and m100) for 4 or 8 weeks. 4-im and 4-m showed immature and mature mice at week 4 after sulfoxaflor administration, respectively. 8-im and 8-m showed immature and mature mice at week 8 after sulfoxaflor administration, respectively. The critical rate is expressed as the *p*-value, and the effect size is expressed as r or η^2^.

Gene	Group (Comparison)	*p*-Value	Effect Size
Pituitary LH	4-im 4-m	0.634 0.946	η^2^ = 0.073 η^2^ = 0.011
	8-im (i0 vs. i100)	0.020 (*p* = 0.02)	η^2^ = 0.508
	8-m (m0 vs. m100)	0.024 (*p* = 0.019)	η^2^ = 0.493
Pituitary FSH	4-im	0.609	η^2^ = 0.079
	4-m	0.523	η^2^ = 0.122
	8-im	0.412	η^2^ = 0.149
	8-m	0.116	η^2^ = 0.324
Star	4-im	0.494	η^2^ = 0.111
	4-m	0.487	η^2^ = 0.123
	8-im	0.300	η^2^ = 0.182
	8-m	0.624	η^2^ = 0.082
P450scc	4-im	0.099	η^2^ = 0.320
	4-m	0.793	η^2^ = 0.041
	8-im	0.137	r = 0.533
	8-m	0.943	η^2^ = 0.011
P450c17	4-im	0.065	η^2^ = 0.365
	4-m	0.469	η^2^ = 0.129
	8-im	0.374	η^2^ = 0.151
	8-m	0.283	η^2^ = 0.205
3β-HSD	4-im	0.122	η^2^ = 0.296
	4-m (m10 vs. m100)	0.022 (*p* = 0.17)	η^2^ = 0.502
	8-im	0.403	r = 0.361
	8-m (m0 vs. m100)	0.018 (*p* = 0.017)	η^2^ = 0.519
17β-HSD	4-im	0.327	η^2^ = 0.170
	4-m	0.577	η^2^ = 0.095
	8-im	0.461	η^2^ = 0.121
	8-m	0.427	η^2^ = 0.143
LHR	4-im	0.076	η^2^ = 0.350
	4-m	0.373	η^2^ = 0.164
	8-im	0.119	η^2^ = 0.298
	8-m	0.376	η^2^ = 0.163
FSHR	4-im	0.263	η^2^ = 0.199
	4-m	0.887	η^2^ = 0.021
	8-im	0.698	r = 0.227
	8-m	0.433	η^2^ = 0.141
Inha	4-im	0.374	η^2^ = 0.151
	4-m (m0 vs. m100)	0.025 (*p* = 0.024)	η^2^ = 0.487
	8-im	0.226	η^2^ = 0.220
	8-m	0.190	η^2^ = 0.261
Shbg	4-im (i0 vs. i10)	0.043 (*p* = 0.036)	η^2^ = 0.408
	4-m	0.077	η^2^ = 0.373
	8-im	0.691	r = 0.227
	8-m	0.069	η^2^ = 0.385
Ki67	4-im	0.107	η^2^ = 0.310
	4-m (m0 vs. m100)	0.029 (*p* = 0.030)	η^2^ = 0.475
	8-im	0.629	η^2^ = 0.074
	8-m	0.506	η^2^ = 0.117
Top2a	4-im	0.492	η^2^ = 0.112
	4-m (m0 vs. m10, m0 vs. m100)	0.002 (*p* = 0.042, 0.001)	η^2^ = 0.678
	8-im	0.300	η^2^ = 0.182
	8-m	0.515	η^2^ = 0.114
Sycp3	4-im	0.799	η^2^ = 0.037
	4-m	0.397	r = 0.377
	8-im	0.773	η^2^ = 0.042
	8-m	0.655	η^2^ = 0.074
Mlh1	4-im	0.220	η^2^ = 0.223
	4-m	0.538	η^2^ = 0.107
	8-im	0.387	η^2^ = 0.146
	8-m	0.659	η^2^ = 0.073
Acr	4-im	0.756	η^2^ = 0.046
	4-m	0.073	η^2^ = 0.378
	8-im	0.398	η^2^ = 0.142
	8-m	0.370	η^2^ = 0.166
Tnp1	4-im	0.416	η^2^ = 0.136
	4-m	0.205	η^2^ = 0.250
	8-im	0.871	η^2^ = 0.023
	8-m	0.374	r = 0.389
Fas	4-im	0.887	η^2^ = 0.020
	4-m	0.205	η^2^ = 0.218
	8-im	0.656	η^2^ = 0.068
	8-m	0.592	r = 0.282
FasL	4-im	0.428	η^2^ = 0.132
	4-m	0.797	η^2^ = 0.040
	8-im	0.468	r = 0.330
	8-m	0.364	η^2^ = 0.394
Bax	4-im	0.753	η^2^ = 0.046
	4-m	0.895	η^2^ = 0.020
	8-im	0.075	r = 0.608
	8-m	0.160	η^2^ = 0.283
Bcl2	4-im	0.368	r = 0.378
	4-m	0.460	η^2^ = 0.132
	8-im	0.465	η^2^ = 0.120
	8-m	0.793	η^2^ = 0.041
Caspase-3	4-im	0.558	η^2^ = 0.093
	4-m	0.270	η^2^ = 0.212
	8-im	0.980	η^2^ = 0.003
	8-m	0.654	r = 0.256
GPx	4-im	0.551	η^2^ = 0.095
	4-m	0.141	η^2^ = 0.300
	8-im	0.395	r = 0.365
	8-m	0.738	r = 0.216
CAT	4-im	0.356	η^2^ = 0.158
	4-m	0.392	η^2^ = 0.157
	8-im	0.498	η^2^ = 0.110
	8-m	0.920	η^2^ = 0.015
SOD1	4-im	0.694	η^2^ = 0.059
	4-m	0.137	η^2^ = 0.304
	8-im	0.512	r = 0.309
	8-m	0.522	r = 0.514
SOD2	4-im (i0 vs. i100, i10 vs. i100)	0.012 (*p* = 0.013, 0.048)	η^2^ = 0.519
	4-m (m0 vs. m100)	0.022 (*p* = 0.018)	η^2^ = 0.501
	8-im	0.504	η^2^ = 0.108
	8-m	0.736	η^2^ = 0.054
Trx1	4-im	0.297	r = 0.417
	4-m	0.380	η^2^ = 0.161
	8-im	0.683	r = 0.233
	8-m	0.137	r = 0.553
Trx2	4-im	0.845	η^2^ = 0.028
	4-m	0.620	η^2^ = 0.083
	8-im	0.515	η^2^ = 0.105
	8-m	0.708	η^2^ = 0.061
TXNIP	4-im	0.944	η^2^ = 0.010
	4-m	<0.001	η^2^ = 0.820
	8-im	0.182	η^2^ = 0.247
	8-m	0.792	η^2^ = 0.042
Nrf2	4-im	0.184	η^2^ = 0.246
	4-m	0.416	η^2^ = 0.147
	8-im	0.567	η^2^ = 0.090
	8-m	0.245	η^2^ = 0.226
OGG1	4-im	0.444	r = 0.341
	4-m	0.062	η^2^ = 0.397
	8-im	0.395	r = 0.365
	8-m	0.180	r = 0.514
ABCD1	4-im	0.553	η^2^ = 0.094
	4-m	0.081	η^2^ = 0.367
	8-im	0.231	η^2^ = 0.217
	8-m	0.411	η^2^ = 0.149
ABCD3	4-im	0.429	η^2^ = 0.131
	4-m (m0 vs. m10, m0 vs. m100)	0.016 (*p* = 0.022, 0.031)	η^2^ = 0.527
	8-im	0.383	η^2^ = 0.148
	8-m	0.621	η^2^ = 0.083
GRP78	4-im	0.997	η^2^ = 0.000
	4-m	0.682	η^2^ = 0.067
	8-im	0.251	η^2^ = 0.206
	8-m	0.650	η^2^ = 0.075
PGC-1α	4-im	0.330	r = 0.398
	4-m	0.585	r = 0.287
	8-im	0.171	η^2^ = 0.255
	8-m (m0 vs. m100)	0.043 (*p* = 0.035)	η^2^ = 0.435
Nrf1	4-im	0.115	η^2^ = 0.303
	4-m	0.554	η^2^ = 0.102
	8-im	0.403	r = 0.361
	8-m	0.590	η^2^ = 0.091
Tfam	4-im (i0 vs. i100)	0.048 (*p* = 0.039)	η^2^ = 0.397
	4-m	0.251	η^2^ = 0.222
	8-im	0.945	η^2^ = 0.009
	8-m	0.733	η^2^ = 0.055
Drp1	4-im	0.928	η^2^ = 0.012
	4-m	0.123	η^2^ = 0.317
	8-im	0.610	η^2^ = 0.079
	8-m	0.651	η^2^ = 0.075
Fis1	4-im (i0 vs. i100)	0.016 (*p* = 0.013)	η^2^ = 0.498
	4-m	0.856	η^2^ = 0.028
	8-im	0.489	r = 0.320
	8-m	0.624	r = 0.269
Mfn1	4-im	0.378	η^2^ = 0.150
	4-m	0.182	η^2^ = 0.267
	8-im	0.641	η^2^ = 0.071
	8-m	0.077	r = 0.629
Opa1	4-im	0.727	r = 0.214
	4-m	0.740	r = 0.215
	8-im	0.382	η^2^ = 0.148
	8-m	0.290	r = 0.436

## Data Availability

The original contributions presented in this study are included in the article/Supplementary Material. Further inquiries can be directed to the corresponding author.

## Supplementary Materials

The following supporting information can be downloaded at: https://www.mdpi.com/article/10.3390/jox15060189/s1. Figure S1: Testicular histology in a wide area of normal and sulfoxaflor-treated mice in immature and mature stages; Figure S2: Testicular histology at low magnification of another testicular area of the normal and sulfoxaflor-treated mice in the immature and mature stages.

## Abbreviations

The following abbreviations are used in this manuscript:

nAChRs	Nicotinic acetylcholine receptors
NOAEL	Non-Observed Adverse Effect Level
LOAEL	Low observable adverse effect level
SFCMS/MS	Supercritical fluid—Mass Spectrometry
Star	Steroidogenic acute regulatory protein
P450scc	Choleserol side-chain cleavage cytochrome P450
P450c17	17 α-hydoroxylase/17, 20 lyase
3β-HSD	3β-Hydroxysteroid dehydrogenase
17-HSD	17β-Hydroxysteroid dehydrogenase
LHR	Luteinizing hormone receptor
FSHR	Follicle-stimulating hormone receptor
InhA	Inhibin-A
Shbg	Sex hormone-binding globulin
Top2a	Topoisomerase 2-alpha
Sycp3	Synaptonemal complex protein 3
Mlh1	MutL homolog 1
Acr	Acrosin
Tnp1	Transition protein 1
FasL	Fas ligand
GPx	Glutathione peroxidase
CAT	Catalase
SOD	Superoxide dismutase
Trx1	Thioredoxin 1
TXNIP	Thioredoxin-interacting protein
Nrf	Nuclear factor E2-related factor
OGG1	8-oxoguanine DNA glycosylase 1
ABCD	Adrenoleukodystrophy protein belongs to the sub-family D
GRP78	Glucose-regulated protein, 78kDa
PGC-1	Peroxisome proliferators-activated receptor-γ co-activator-1α
Tfam	Mitochondrial transcription factor A
Drp1	Dynamin-related protein 1
Fis1	Mitochondrial fission 1 protein
Mfn1	Mitofusin 1
Opa1	Optic atrophy protein 1
LH	Luteinizing hormone
FSH	Follicle stimulating hormone
IL	interleukin
