# Supplementary material for: Low Concentrations of Sulfoxaflor Do Not Adversely Affect mRNA Levels in Various Testicular Cells When Administered to Either Mature or Immature Mice
Source: J Xenobiot. 2025 Nov 7;15(6):189. doi: 10.3390/jox15060189 (PMC12641737; doi:10.3390/jox15060189)
Supplement: Supplementary file 1 [file jox-15-00189-s001.zip › jox-3894651-supplementary.pdf]

Low concentrations of sulfoxaflor do not adversely affect mRNA levels in various testicular cells when administered to either mature or immature mice

Hayato Terayama, Kenta Nagahori, Daisuke Kiyoshima, Tsutomu Sato, Yoko Ueda, Masahito Yamamoto, Kaori Suyama, Tomoko Tanaka, Midori Yamamoto, Akifumi Eguchi, Emiko Todaka, Kenichi Sakurai, Shogo Hayashi, Haruki Yamada and Kou Sakabe

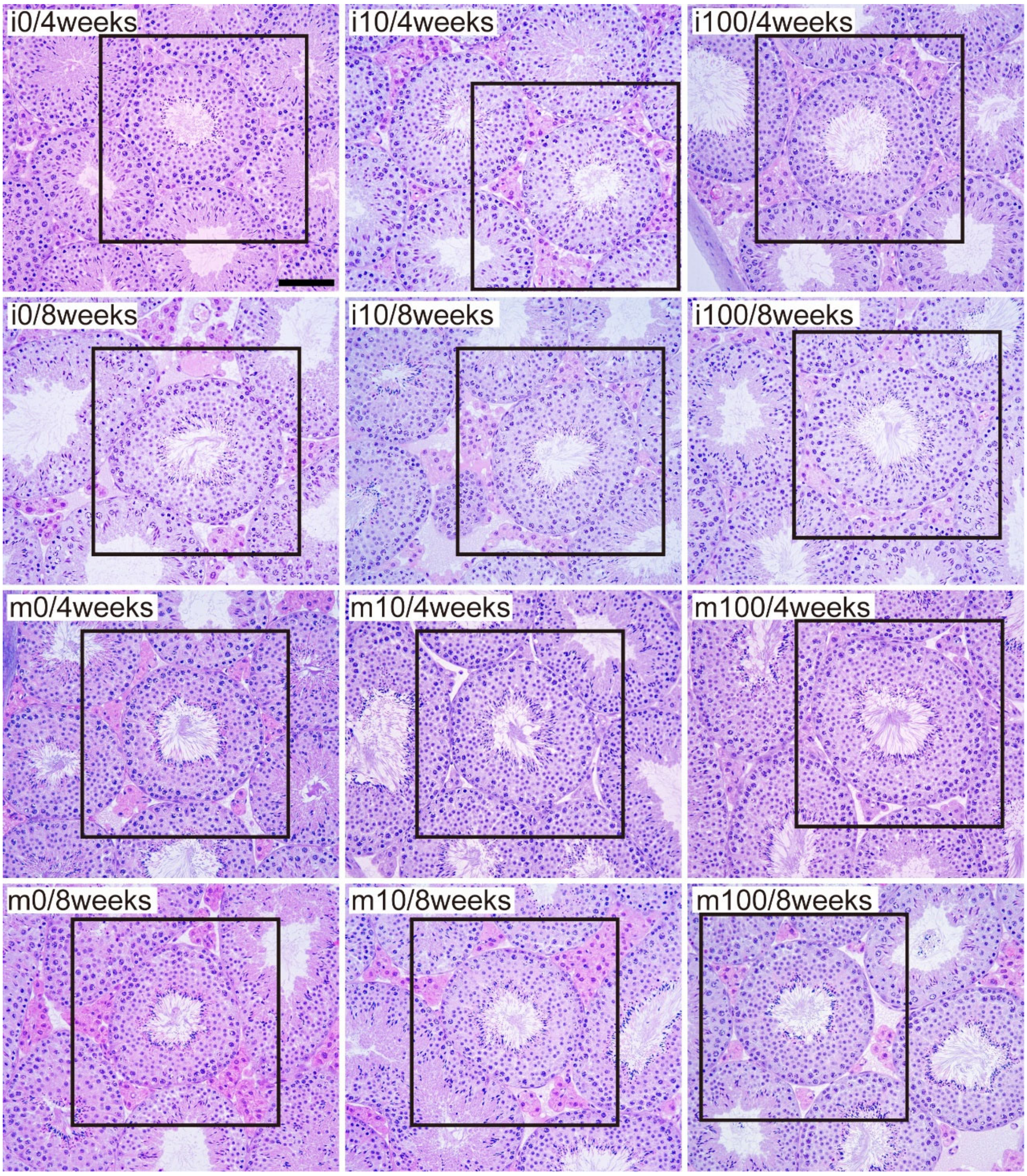

Figure S1: Testicular histology in a wide area of normal and sulfoxaflor-treated mice in immature and mature stages.

Mice aged 3 weeks (i0, i10, and i100) and 8 weeks (m0, m10, and m100) were exposed to normal water (i0 and m0), sulfoxaflor 10 mg/kg (i10 and m10), and sulfoxaflor 100 mg/kg (i100 and m100) for 4 and 8 weeks. Seminiferous tubules (Figure 2) shown in the manuscript are indicated by squares (□). Scale bar: 100 μm

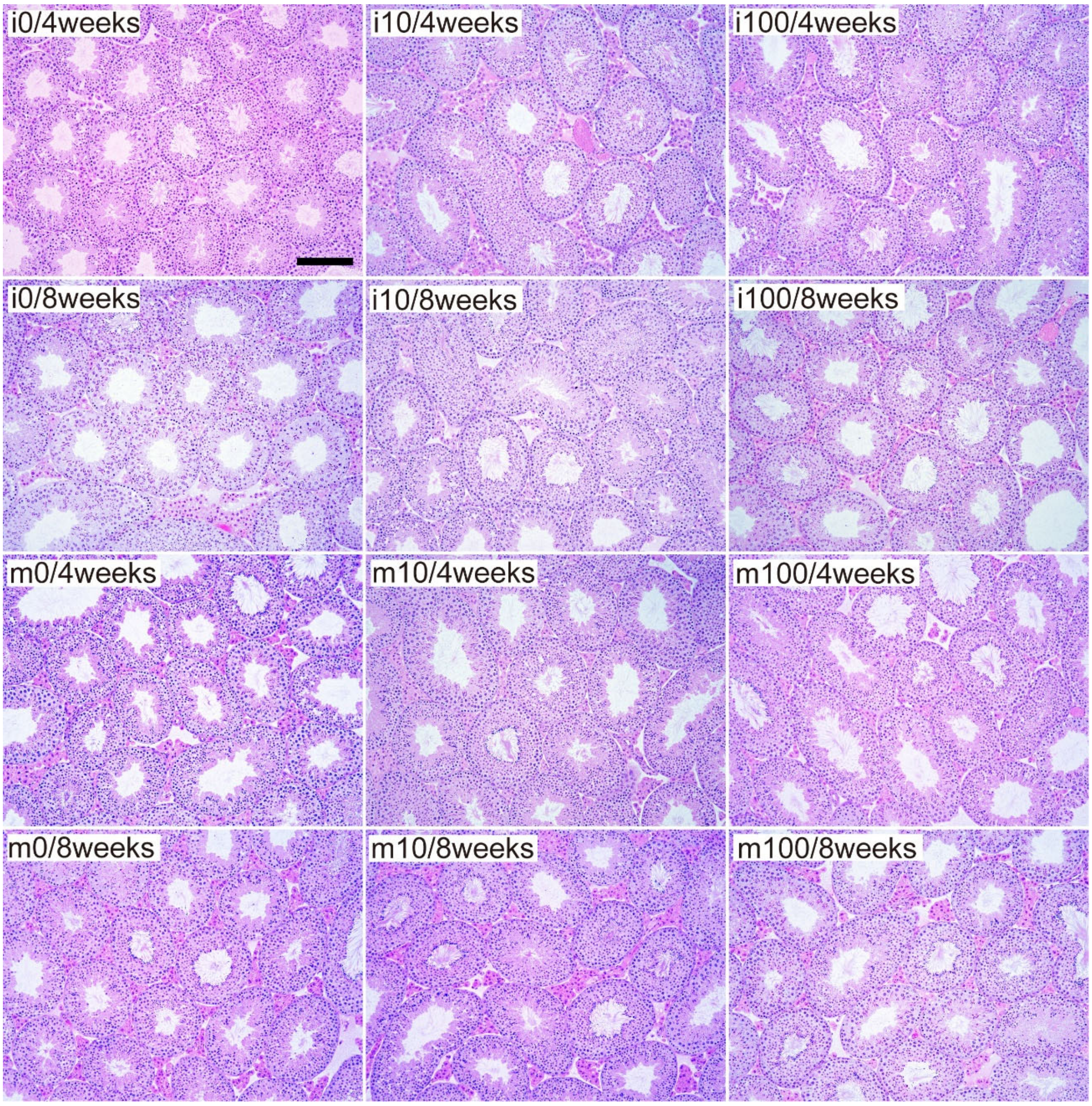

Figure S2: Testicular histology at low magnification of another testicular area of the normal and sulfoxaflor-treated mice in the immature and mature stages.

Mice aged 3 weeks (i0, i10, and i100) and 8 weeks (m0, m10, and m100) were exposed to normal water (i0 and m0), sulfoxaflor 10 mg/kg (i10 and m10), and sulfoxaflor 100 mg/kg (i100 and m100) for 4 and 8 weeks. Scale bar: 200 μm
